# Sleep and Sleep Disorder Knowledge Among Physicians Working in Qatar’s Primary Health Care Corporation: A Cross-Sectional Study

**DOI:** 10.3390/clockssleep8010013

**Published:** 2026-03-12

**Authors:** Mohamed Salem, Fawzia Alhor, Amr Ouda, Soha Halawa, Yara Abuazab, Ibrahim Elmakaty

**Affiliations:** 1 Family Medicine Department, Primary Health Care Corporation, Doha P.O. Box 26555, Qatar; 2 Family Medicine Residency Program, Department of Medical Education, Hamad Medical Corporation, Doha P.O. Box 3050, Qatar

**Keywords:** cross-sectional studies, education, medical, physicians, primary care, primary health care, Qatar, sleep, surveys and questionnaires

## Abstract

Sleep is a fundamental biological process essential for physical, cognitive, and mental health, yet sleep disorders remain underrecognized in primary care. Given the central role of primary care physicians (PCPs) in early identification and management, this study aimed to assess sleep and sleep disorder knowledge among PCPs working within the Primary Health Care Corporation in Qatar. A cross-sectional study was conducted using the validated 30-item Assessment of Sleep Knowledge in Medical Education (ASKME) questionnaire, alongside demographic and clinical practice variables. The primary outcomes were the overall ASKME percentage score and participants achieving adequate sleep knowledge (≥60%). A total of 110 PCPs were included in the analysis. The mean overall ASKME score was 56.5%, and 44.5% of participants achieved adequate sleep knowledge. Knowledge was highest in circadian sleep–wake regulation and basic sleep principles, and lowest in common sleep disorders, sleep architecture, and the effects of drugs and alcohol on sleep. In multivariable logistic regression, years of clinical experience was the only factor independently associated with adequate sleep knowledge. These findings indicate persistent gaps in clinically relevant sleep knowledge among PCPs and underscore the need for targeted sleep education within primary care to support early and effective management of sleep disorders. However, the achieved sample size was substantially smaller than the initially calculated target of 260, limiting statistical power; therefore, the non-significant findings may reflect a Type II error, and the regression analyses should be interpreted with caution.

## 1. Introduction

Sleep is a naturally recurring biological state characterized by reduced responsiveness to external stimuli, relative inactivity, and distinct neurophysiological patterns. It is universally recognized as a fundamental biological necessity essential for physical restoration, cognitive performance, emotional regulation, and overall health because of its role in metabolic, cardiovascular, immune, and neural processes [1]. Major health organizations, including the American Academy of Sleep Medicine, the World Health Organization, and the National Institutes of Health, emphasize sleep’s centrality to health, positioning it alongside nutrition and exercise as a primary determinant of well-being [1].

Sleep regulation is governed by the interplay of circadian rhythms and homeostatic processes that synchronize physiological functions with environmental cues, particularly the light–dark cycle [2]. The circadian timing system coordinates daily rhythms in hormonal release and sleep–wake timing, whereas sleep–wake homeostasis reflects pressure to sleep that accumulates during prolonged wakefulness [3]. Disruptions in these regulatory mechanisms are linked with impaired sleep and adverse health consequences [4,5], underscoring the biological complexity of sleep beyond simple behavioral rest.

Normal sleep architecture consists of cyclic transitions between non-rapid eye movement (NREM) and rapid eye movement (REM) stages, typically occurring in approximately 90 min cycles throughout the night [6]. NREM sleep includes stages associated with deeper restorative functions, whereas REM sleep features cortical activation and dreaming. Each stage serves distinct physiological roles, including memory consolidation and autonomic regulation, and deviations from these patterns are integral to many sleep disorders [5].

Healthy sleep embodies adequate duration, continuity, regularity, and quality and is associated with favorable cognitive, emotional, and physical health outcomes. Insufficient or disrupted sleep adversely affects cardiovascular, metabolic, and neurocognitive systems, contributing to increased risk of hypertension, diabetes, mood disorders, stroke, and impaired immune function [1,4,5]. Accordingly, recent frameworks propose that sleep health should be considered a multidimensional construct with implications beyond the absence of sleep disorders, embracing efficiency, timing, and alignment with circadian rhythms [7].

Sleep disorders, including insomnia, sleep-related breathing disorders, circadian rhythm abnormalities, hypersomnolence, and parasomnias, are highly prevalent yet frequently underrecognized in clinical settings [5]. In the general adult population, epidemiological evidence suggests that a substantial proportion of individuals experience chronic insufficient sleep and clinically relevant sleep problems, with estimates in the United States indicating that more than one-third of adults achieve less than the recommended duration of sleep and that an estimated 50–70 million adults have a diagnosable sleep disorder [5].

The Assessment of Sleep Knowledge in Medical Education (ASKME) questionnaire is a validated instrument developed to objectively assess clinicians’ knowledge of sleep physiology and sleep disorders. It evaluates multiple core domains of sleep medicine and demonstrates strong psychometric properties, including high internal consistency and good discriminant validity across diverse clinician populations [8]. Owing to its methodological rigor and reproducibility, the ASKME questionnaire has been widely adopted in studies assessing sleep knowledge among healthcare professionals.

A previous cross-sectional study in Qatar assessed physicians’ knowledge of sleep disorders using the validated ASKME questionnaire in a tertiary hospital setting and reported generally low levels of sleep medicine knowledge. Fewer than 40% of respondents achieved the predefined adequate sleep knowledge threshold (≥60%), with a mean score of 15.53 out of 30 (51.76%), and knowledge did not differ significantly by years of training or professional rank, suggesting persistent educational gaps despite advancing clinical experience. However, that study included hospital-based physicians across multiple specialties and may not reflect the knowledge needs or clinical responsibilities of physicians practicing in primary care.

In Qatar, the Primary Health Care Corporation (PHCC) serves as the main entry point for patients with sleep-related complaints, where initial recognition, counseling, and referral decisions are typically made. Accordingly, an updated assessment within the PHCC is needed to establish a contemporary baseline specific to the primary care context and to guide targeted educational priorities relevant to frontline practice. Since the abovementioned hospital-based assessment, Qatar’s healthcare context has evolved considerably. Rapid population growth, increasing prevalence of cardiometabolic risk factors associated with sleep disorders, and expansion of the PHCC as the primary provider of frontline care have increased the likelihood that sleep-related complaints are first encountered in primary care. In this setting, physicians play a critical role in early recognition, counseling, and referral, making sleep medicine knowledge particularly consequential. These contextual developments further support the need for a contemporary, primary care-focused reassessment of sleep knowledge in Qatar rather than reliance on older data derived from tertiary care settings.

In primary care, physician knowledge of sleep physiology and sleep disorders can directly influence clinical practice. Limited knowledge may lead to inadequate screening for conditions such as insomnia and obstructive sleep apnea, misattribution of sleep-related symptoms to other medical or psychiatric disorders, delayed referral to sleep specialists, and suboptimal initial management [5]. As primary care physicians are typically the first point of contact for patients with sleep complaints, deficiencies in sleep medicine knowledge may influence care pathways and contribute to prolonged symptom burden or missed opportunities for early intervention. Given the central role of PCPs in the identification and management of sleep disorders, evaluating sleep-related knowledge within Qatar’s PHCC is therefore essential.

Accordingly, this study used the validated ASKME questionnaire to assess overall and domain-specific knowledge of sleep and sleep disorders among PCPs working within the PHCC. The study aims to identify specific knowledge gaps and explore professional and educational factors associated with knowledge levels. Importantly, the study evaluates physician knowledge rather than clinical decision-making or patient outcomes, and the findings are intended to inform targeted educational strategies and future workforce development initiatives within Qatar’s primary care system.

## 2. Results

### 2.1. Responses and Participant Flow

**Figure 1:**
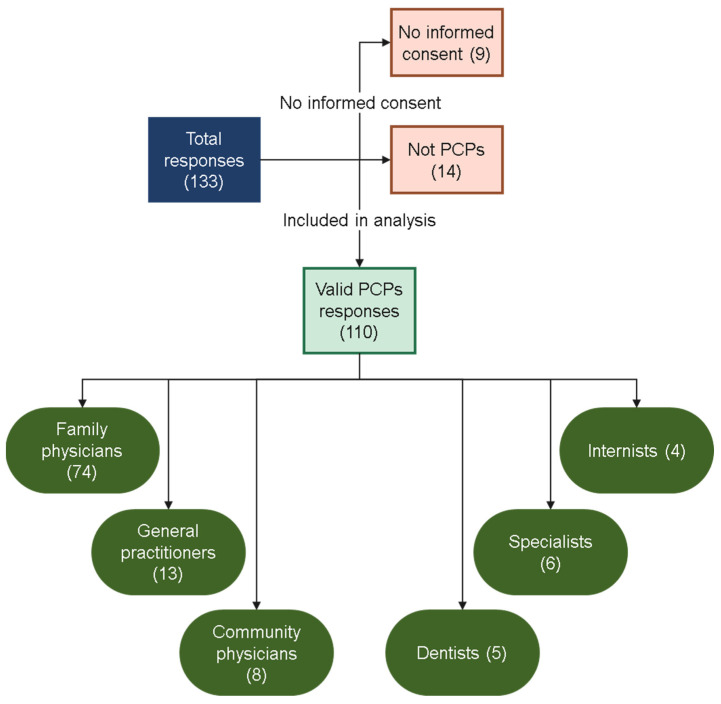
Flowchart of study participant recruitment.

A total of 133 responses were received via Microsoft Forms. Nine respondents did not provide informed consent and were therefore excluded. Additionally, 14 responses were excluded because they were not from PCPs (pharmacists (n = 7), nurses (n = 2), optometrists (n = 2), lab technologists (n = 2), physiotherapist (n = 1)). Consequently, 110 valid responses were included in the final analysis, corresponding to approximately 13.75% of the estimated PHCC primary care physician workforce (≈800 physicians). A flowchart of participant recruitment for this study is presented in Figure 1. Among the included respondents, most were family medicine physicians (n = 74), followed by general practitioners (n = 13), community medicine physicians (n = 8), dentists (n = 5), specialists (n = 6), and internal medicine physicians (n = 4).

### 2.2. Participant Characteristics

The demographic and professional characteristics of the 110 included participants are summarized in Table 1. Most respondents were aged between 40 and 59 years (n = 76, 69.1%), with a predominance of females (60.9%). Participants represented diverse national backgrounds, including Qatari physicians (20.0%), individuals of other Arab nationalities (24.6%), and individuals from Western countries (21.8%), while 32.7% preferred not to disclose their nationality.

The majority of participants obtained their primary medical degree outside Qatar, most commonly from other Arab countries (27.3%) or South Asia (12.7%). Family medicine physicians constituted the largest specialty group (67.3%). Nearly two-thirds of respondents reported providing sleep-related counseling as part of their clinical practice (63.6%), although fewer than half had received formal sleep training (40.0%). Notably, 41.8% reported never having updated their sleep knowledge, and only 21.8% had previously attended sleep-related continuing professional development (CPD) activities.

### 2.3. Item-Level Sleep Knowledge Performance

Knowledge performance varied considerably across items. High proportions of correct responses were observed for items related to circadian physiology and clinical relevance, such as nocturnal melatonin secretion (87.3%), the association between inadequate sleep and hyperactivity in children (85.5%), and the role of weight loss in managing mild obstructive sleep apnea (86.4%). The ASKME instrument demonstrated acceptable internal consistency in our sample (Cronbach’s α = 0.76).

Conversely, substantial knowledge gaps were identified in several clinically relevant areas. Fewer than one-quarter of participants correctly answered items related to narcolepsy epidemiology (21.8%), slow-wave-sleep distribution throughout the night (23.6%), and the neurobiological basis of narcolepsy (25.5%). Knowledge regarding the effects of alcohol on sleep regulation was also limited, with only 18.2% correctly identifying sleep recovery patterns in individuals with alcohol use disorder.

### 2.4. Domain-Specific and Overall Sleep Knowledge Scores

Domain-specific and overall ASKME scores are summarized in Table 2. Participants achieved the highest mean scores in the domains of circadian sleep–wake control (mean 72.9%, standard deviation [SD] 22.3) and basic sleep principles (mean 63.6%, SD 22.1). In contrast, lower performance was observed in domains related to common sleep disorders (mean 46.9%, SD 19.3), normal sleep architecture (mean 49.7%, SD 23.9), and the effects of drugs and alcohol on sleep (mean 49.1%, SD 29.9).

The overall mean percentage score was 56.5% (SD 15.9; range 10.0–96.7). A total of 49 participants (44.5%) achieved a score of ≥60%, meeting the predefined threshold for adequate sleep knowledge. Assessment of score distribution demonstrated approximate normality. Visual inspection of the histogram with an overlaid density curve showed a bell-shaped distribution, and the Shapiro–Wilk test confirmed no significant deviation from normality (*p* = 0.304).

### 2.5. Comparison of Sleep Knowledge Across Participant Characteristics

Beyond descriptive comparisons, inferential analyses were conducted to evaluate associations between participant characteristics and overall sleep knowledge scores. Comparisons of mean ASKME scores across demographic and clinical practice characteristics are presented in Table 3. Mean sleep knowledge scores did not differ significantly by age group (one-way analysis of variance [ANOVA], *p* = 0.340), gender (independent-samples *t*-test, *p* = 0.859), nationality (*p* = 0.233), specialty (*p* = 0.755), degree level (*p* = 0.330), provision of sleep-related counseling (*p* = 0.746), formal sleep training (*p* = 0.231), or attendance at sleep-related CPD activities (*p* = 0.292).

Years of clinical experience showed a strong association with sleep knowledge; because the assumption of homogeneity of variances was violated (Bartlett’s test *p* = 0.021), Welch’s ANOVA was applied and demonstrated a significant overall effect (*p* = 0.0015). Post hoc Games–Howell comparisons revealed that participants with more than 20 years of experience had significantly higher scores compared with those with 1–5 years, 6–10 years, and 11–15 years of experience.

Mean scores also differed significantly according to the recency of sleep knowledge updates (one-way ANOVA, *p* = 0.033). Post hoc Tukey testing indicated that participants who had updated their sleep knowledge within the past 1–2 years scored significantly higher than those who reported never updating their sleep knowledge.

### 2.6. Factors Associated with Adequate Sleep Knowledge (≥60%)

Multivariable logistic regression was used to analytically identify factors independently associated with achieving adequate sleep knowledge. Several effect estimates were associated with wide confidence intervals, indicating limited precision, and these results should therefore be interpreted cautiously. The results of the univariable and multivariable logistic regression analyses are presented in Table 4. In the univariable analyses, a higher age category (odds ratio [OR] 1.87, 95% confidence interval [CI] 1.20–2.93), more years of experience (OR 1.86, 95% CI 1.36–2.56), and more recent sleep knowledge updates (OR 1.31, 95% CI 1.03–1.67) were significantly associated with achieving adequate sleep knowledge. Participants who had obtained a medical degree from a Western country also exhibited higher odds of adequate sleep knowledge compared with Qatari-trained physicians (OR 6.67, 95% CI 1.14–38.83).

In the multivariable model adjusting for gender, years of experience, recency of sleep knowledge update, and attendance at sleep-related CPD activities, after adjustment for potential confounders, years of clinical experience remained the only factor independently associated with achieving adequate sleep knowledge (adjusted OR 1.79, 95% CI 1.28–2.49, *p* = 0.001). Gender, recency of knowledge update, and CPD attendance were not independently associated with adequate sleep knowledge after adjustment. Other demographic, educational, and practice-related variables were not independently associated with adequate sleep knowledge after adjustment. Given the limited number of outcome events, regression estimates should be interpreted with caution, and emphasis is placed on effect sizes and confidence intervals rather than point estimates alone.

## 3. Discussion

### 3.1. Principal Findings

This cross-sectional study provides an in-depth evaluation of sleep and sleep disorder knowledge among PCPs within Qatar’s PHCC and reveals several important findings. Overall, sleep knowledge was suboptimal, with a mean ASKME score of 56.5% and fewer than half of participants achieving the predefined threshold for adequate sleep knowledge (≥60%). This indicates that substantial knowledge gaps may be present among PCPs despite their central role in the frontline identification and management of sleep-related conditions. These findings should be interpreted as reflecting knowledge gaps rather than direct measures of clinical performance, as actual screening, referral, and treatment practices were not assessed in this study. Given the study’s sample size and voluntary sampling design, these findings should be interpreted as indicative patterns within the PHCC physician population rather than precise estimates of sleep knowledge levels or determinants.

Knowledge performance varied markedly across ASKME domains. Participants demonstrated relatively stronger understanding of circadian sleep–wake regulation and basic sleep principles, reflecting familiarity with foundational concepts of sleep physiology. In contrast, performance was consistently weaker in domains more directly related to clinical decision-making, including common sleep disorders, normal sleep architecture, and the effects of drugs and alcohol on sleep. These gaps are clinically meaningful, as deficiencies in these areas may hinder timely recognition, appropriate counseling, and initial management of prevalent conditions such as insomnia, obstructive sleep apnea, and substance-related sleep disturbances.

Within this sample, years of clinical experience was the factor most consistently associated with higher sleep knowledge scores. In both unadjusted and multivariable analyses, greater experience was independently associated with higher odds of achieving adequate sleep knowledge, whereas formal sleep training and attendance at sleep-related CPD activities were not independently predictive after adjustment. This apparent paradox warrants careful interpretation. One possible explanation is that formal sleep training and CPD exposure may be heterogeneous in content, duration, and quality, with limited emphasis on clinically actionable knowledge or insufficient reinforcement over time.

Another possibility is that cumulative clinical exposure, repeated patient encounters, and informal learning through practice may play a larger role in consolidating sleep-related knowledge than episodic educational activities. Self-selection and recall bias may also contribute, as physicians with a pre-existing interest in sleep medicine may be more likely to seek additional training yet not necessarily achieve higher objective knowledge scores, or may overestimate prior training exposure. Additionally, formal sleep education may have occurred earlier in training and not been retained without continued clinical application. Together, these findings suggest that current training pathways may lack sufficient depth, consistency, or longitudinal reinforcement in sleep medicine, underscoring the need for structured, competency-based approaches rather than reliance on isolated educational exposures.

### 3.2. Interpretation of Results and Comparison with the Existing Literature

The present study demonstrates that sleep knowledge among PCPs in Qatar remains suboptimal, a finding that is consistent with a growing body of international literature using the ASKME instrument and reinforcing concerns that sleep medicine continues to be underrepresented in clinical training despite its growing public health relevance. These findings align closely with prior studies conducted across the Middle East, Europe, Africa, and Asia, which consistently report low baseline sleep knowledge among physicians and trainees.

Our results closely mirror those of the only previously published ASKME-based study conducted in Qatar at a tertiary care hospital of the Hamad Medical Corporation. In that study, only 36.1% of physicians achieved adequate sleep knowledge, with no significant differences by rank or years of training [9]. While the Hamad hospital study included residents, fellows, and consultants across multiple specialties, the present study extends these findings to the primary care setting, where early detection of sleep disorders is most critical. Together, these studies suggest that insufficient sleep knowledge is a systemic issue across levels of care in Qatar rather than being confined to hospital-based practice.

Comparable deficits have been reported among primary care physicians in Saudi Arabia, where Saleem et al. found a mean ASKME score of 48% and demonstrated that fewer than 40% of PCPs routinely referred patients with suspected sleep disorders [10]. Similarly to our findings, sleep knowledge in that cohort was not associated with gender or formal qualifications, highlighting a persistent gap in structured sleep medicine education within primary care training pathways. These regional parallels are particularly relevant given the similarities in healthcare systems and referral structures across Gulf countries.

Studies conducted among medical students and early-career physicians further contextualize our findings by illustrating that sleep knowledge deficits begin early in medical education and often persist into clinical practice. Research from Saudi Arabia, Lebanon, Nigeria, Jordan, and India using the ASKME questionnaire has consistently shown that fewer than 10–30% of participants achieve adequate sleep knowledge [11,12,13,14,15]. Notably, the mean scores reported in these studies are comparable to those observed in our cohort, suggesting that deficiencies in sleep medicine education are neither country-specific nor confined to resource-limited settings.

Across studies, performance tends to be higher in domains related to basic sleep principles and circadian regulation, with consistently poorer performance in clinically actionable domains such as common sleep disorders, sleep architecture, and the effects of medications and substances on sleep. This domain-specific pattern observed in our study has also been reported among medical students in Saudi Arabia and Nigeria and among dental interns in Jordan [11,14,16]. These findings are particularly concerning, as deficiencies in these domains directly affect diagnostic accuracy, patient counseling, and referral decisions in primary care.

An important finding of the present study is that years of clinical experience were independently associated with higher odds of adequate knowledge. This contrasts with interventional studies demonstrating that even brief educational interventions can significantly improve ASKME scores among medical students [15,17]. The discrepancy suggests that while structured education is effective when implemented, such training may be insufficiently comprehensive, inconsistently delivered, or inadequately reinforced in routine CPD for practicing physicians.

Although multiple studies using the ASKME instrument have consistently demonstrated suboptimal sleep knowledge, reported determinants of knowledge and the magnitude of deficits vary considerably across settings. Some studies have found associations with level of training or professional rank, whereas others (including the present study) did not observe independent effects of formal training or qualifications. These inconsistencies likely reflect differences in study populations (e.g., medical students, residents, hospital-based physicians, or primary care physicians), as well as heterogeneity in healthcare systems, exposure to sleep-related clinical encounters, and the content and quality of sleep medicine education.

Methodological differences may also contribute to variability in findings, including differences in sample size, response rates, handling of missing data, and analytic approaches to continuous versus dichotomized ASKME scores. In addition, the clinical relevance of sleep knowledge may differ by practice setting; in primary care, experiential learning through repeated patient encounters may exert a stronger influence on knowledge acquisition than formal training, whereas structured education may play a larger role earlier in training. Together, these factors suggest that inconsistencies across prior studies are not contradictory but context-dependent, underscoring the importance of interpreting ASKME findings within the specific healthcare and training environment being studied. These comparisons should be interpreted cautiously, as differences in sampling strategies, healthcare settings, and study power limit the extent to which direct inferences can be drawn.

### 3.3. Implications for Clinical Practice, Education, and Policy

These findings have potential implications for primary care practice and health system planning in Qatar. As most patients with sleep-related complaints initially present to primary care, gaps in physicians’ sleep-related knowledge may represent one factor that could contribute to under-recognition, delayed diagnosis, or suboptimal initial management of common sleep disorders. However, as this study assessed knowledge rather than observed clinical behavior, these implications should be interpreted cautiously and viewed as indicative of possible areas of vulnerability in care delivery rather than direct evidence of practice deficiencies.

From an educational perspective, the domain-specific knowledge gaps identified in this study suggest that more targeted educational approaches, rather than broad or ad hoc exposure, may be beneficial. At the undergraduate level, sleep medicine content could be more explicitly aligned with existing physiology and clinical teaching blocks, with clearly defined learning objectives addressing clinically relevant topics such as normal sleep architecture, insomnia, obstructive sleep apnea, and medication-related sleep disturbances. Reinforcement through case-based discussions within internal medicine, family medicine, and pediatrics rotations may help contextualize foundational knowledge within routine clinical scenarios.

Within family medicine residency training, sleep medicine competencies could be further supported through structured, case-based teaching focused on common primary care presentations, alongside clear guidance on initial management and referral pathways. Incorporating sleep-related scenarios into workplace-based assessments or OSCE stations may offer opportunities to evaluate applied knowledge, while recognizing that such educational strategies require further evaluation.

At the level of CPD within the PHCC, focused CPD activities may help address practical aspects of sleep assessment and counseling relevant to primary care workflows. Given the observed association between greater clinical experience and higher knowledge scores in this study, early-career physicians may represent a particularly relevant target group for future educational initiatives. From a policy perspective, these findings may help inform future discussions regarding the integration of core sleep medicine competencies within PHCC training and CPD frameworks, while acknowledging that further research in larger and more representative samples is needed before drawing firm conclusions or implementing system-level changes.

### 3.4. Strengths and Limitations

This study has several strengths. It is the first, to our knowledge, to comprehensively assess sleep knowledge among PCPs in Qatar using a validated instrument, and it includes a diverse sample reflecting real-world primary care practice. The use of the ASKME questionnaire allows direct comparison with international studies and prior local data, enhancing the interpretability and relevance of the findings.

Nevertheless, several limitations should be acknowledged. The primary limitation of this study is the relatively small achieved sample size compared with the initially calculated minimum requirement. Although the questionnaire was distributed institution-wide, only 110 physicians participated, which may have limited the statistical power to detect true associations. Consequently, the absence of statistically significant relationships for several variables should be interpreted cautiously, as these findings may reflect a Type II error rather than a true lack of association. In addition, the multivariable logistic regression model should be interpreted with caution, as the limited number of outcome events relative to the number of predictors increases the risk of overfitting and unstable effect estimates. It is also important to note that the events-per-variable ratio in the multivariable logistic regression model was relatively low, which may increase the risk of model overfitting and unstable parameter estimates. Although a post hoc power analysis was considered, it was not performed, because such analyses provide little information beyond the observed effect estimates and confidence intervals and may be misleading when interpreted after results are known. Instead, the potential impact of limited statistical power is addressed through cautious interpretation of null findings, explicit acknowledgment of a possible Type II error, and reporting of effect estimates with confidence intervals.

The cross-sectional design precludes causal inference, and participation was voluntary, introducing the possibility of selection bias. Additionally, sleep knowledge was assessed using a self-administered questionnaire, which may not fully capture clinical competence or actual practice behaviors. In addition, years of clinical experience were collected in categorical form, which limited the ability to model this variable as a continuous predictor and may have reduced statistical efficiency compared with interval-level measurement. Furthermore, the non-random sampling used might introduce volunteer bias as physicians interested in sleep may have been more inclined to participate. A comparison between responders and non-responders was not possible because data collection was conducted anonymously.

The study was conducted within a single national primary care system, which may limit generalizability to other healthcare settings. Physicians practicing outside the public PHCC system were not included, and the findings may not be generalizable to private-sector or hospital-based physicians in Qatar. A further limitation is that the response rate could not be calculated because the questionnaire was distributed centrally by the institutional research department, and the total number of recipients was not available to the investigators.

## 4. Materials and Methods

### 4.1. Study Design

This study was designed as a descriptive cross-sectional study using a self-administered questionnaire for data collection. To ensure scientific rigor and transparent reporting, the study adhered to the Strengthening the Reporting of Observational Studies in Epidemiology (STROBE) checklist [18].

### 4.2. Settings

The study was conducted within the PHCC in Qatar. The PHCC is Qatar’s government-run organization responsible for delivering public primary healthcare services nationwide through a network of health centers. Physicians practicing outside the PHCC, including those in private clinics or hospital-based settings, are not employed by the PHCC and were therefore outside the institutional scope of this study.

Data were collected over a period of approximately five months, from 29 June 2025 to 1 January 2026. The questionnaire was administered electronically using Microsoft Forms (Microsoft Corporation, Redmond, WA, USA). Invitations containing the questionnaire link and detailed study information were distributed to eligible PCPs via their official corporate email accounts by the PHCC research department.

### 4.3. Participants and Sampling

In the Qatari healthcare system, physicians practicing within the PHCC constitute the primary care workforce and provide first-contact care regardless of their original specialty training. Accordingly, all physicians working clinically within PHCC health centers, including family medicine physicians and other medical specialists providing primary care services, were considered eligible. Eligible participants included PCPs practicing regularly within PHCC health centers, including general practitioners, family medicine specialists, and consultants. Subspecialty physicians working within the PHCC, such as pediatricians, ophthalmologists, dermatologists, and dentists, were also included. Non-physician healthcare staff (e.g., nurses, dietitians, physiotherapists), as well as interns and residents, were excluded. The questionnaire was distributed via official PHCC corporate email lists to all eligible physicians employed across all PHCC health centers in Qatar. No sampling of health centers or individual physicians was undertaken. Participation was voluntary, and the final sample therefore represents a voluntary response subset of the PHCC physician population. Data collection continued until no new responses were received over a sustained period.

### 4.4. Sample Size Calculation

The required sample size was estimated using Cochran’s formula for cross-sectional studies [19]. Assuming a 95% confidence level (Z = 1.96), a margin of error of 5% (d = 0.05), and maximum variability (*p* = 0.5), and adjusting for a finite population of approximately 800 PCPs within the PHCC, the calculated minimum sample size was 260 participants. However, due to the historically low response rates for institutional questionnaires, data collection was continued over an extended period and concluded when response saturation was reached and no additional responses were received.

### 4.5. Collected Variables and Questionnaire Instrument

The questionnaire consisted of two components. The first component collected demographic and professional characteristics, including age, gender, nationality, years of clinical experience, and highest medical qualifications. Clinical practice characteristics related to sleep medicine were also collected, including provision of sleep-related counseling, prior formal sleep training, timing of the most recent sleep knowledge update, and attendance at sleep-related CPD activities. For selected demographic variables that could potentially increase identifiability (e.g., nationality, country of medical degree), a “Prefer not to say” response option was provided to protect participant anonymity, in accordance with institutional ethical guidance. For variables not considered identifiable, responses were mandatory and did not include a “Prefer not to say” option. In analyses involving variables with a “Prefer not to say” option, these responses were excluded from comparative and regression analyses, as they do not constitute a meaningful analytical category. Such responses were retained only in descriptive summaries to ensure transparent reporting.

The second component consisted of the self-administered ASKME questionnaire, used in its original form without modification [8]. The ASKME instrument was developed in 2001 as a standardized tool to assess sleep medicine knowledge in medical education, addressing the lack of previously validated and widely adopted measures in this field. Instrument development was conducted over four phases: initial item selection based on a comprehensive literature review and existing sleep curricula; an expert panel review to establish face validity and educational relevance; pilot testing for reliability and construct validity; and final item selection.

During development, sleep knowledge content was initially organized into six domains: basic sleep principles, circadian sleep–wake regulation, normal sleep architecture, common sleep disorders, medical and psychiatric illness and sleep, and the effects of drugs and alcohol on sleep. Following item analysis and validation procedures, questions related to medical and psychiatric illness and sleep were excluded due to poor discriminant validity, resulting in a final 30-item instrument covering five domains. All items are formatted as “True,” “False,” or “Don’t know,” with the latter included to reduce random guessing. Each correct response is awarded one point, yielding a total score ranging from 0 to 30, commonly expressed as a percentage.

In the original validation study, the ASKME questionnaire demonstrated strong psychometric properties, including high internal consistency reliability (Kuder–Richardson Formula 20 [which is a measure of internal consistency reliability for dichotomous items and represents a special case of Cronbach’s alpha when items are scored 0/1] coefficient = 0.89) [8]. Furthermore, the ASKME questionnaire demonstrated good discriminant validity by yielding significantly different knowledge scores across groups with varying levels of training and expertise, including accredited sleep specialists, practicing physicians, medical students, and nurses [8]. Consistent with prior the original ASKME-based report, a cutoff of ≥60% correct responses was used to indicate a higher level of sleep knowledge.

### 4.6. Data Collection Procedure

Questionnaire distribution was conducted by the PHCC Research Department using centralized corporate email lists. Because questionnaire distribution was performed using centralized institutional email lists, some non-physician staff received the questionnaire link. Data were collected electronically over a six-month period. The initial questionnaire invitation was distributed on 29 June 2025 via the official PHCC corporate email account. Two reminder emails were subsequently sent to eligible physicians on 27 July 2025 and 3 September 2025 to enhance participation. Data collection was formally closed on 1 January 2026. The investigators did not have access to the distribution lists or to the exact number of physicians who received the questionnaire link. Data were collected online using the Microsoft Forms platform. Participation was voluntary, and responses were collected anonymously. The questionnaire required approximately 10–15 min to complete.

### 4.7. Outcomes of Interest

The primary outcomes of interest were the overall mean percentage score on the ASKME questionnaire (continuous outcome) and the proportion of physicians achieving adequate sleep knowledge as defined by a score of ≥60% (binary outcome). The cutoff score of ≥60% for adequate sleep knowledge, which has been used in prior ASKME-based studies, was selected in our study to facilitate comparison across studies and to identify relatively higher levels of sleep knowledge [9,11,14]. Secondary outcomes included domain-specific sleep knowledge scores across the five ASKME domains and the association between demographic and clinical practice characteristics and sleep knowledge outcomes.

### 4.8. Statistical Analysis

Demographic and clinical practice characteristics were summarized using frequencies and percentages. Correct response rates for individual ASKME items and mean scores for each domain were presented descriptively. Incomplete questionnaires were excluded from the final analysis, and no imputation for missing data was performed.

The overall ASKME percentage score was treated as a continuous variable and assessed for normality using the Shapiro–Wilk test and visual inspection of histograms with density curves [20]. Independent-samples *t*-tests and one-way ANOVA were used to compare mean scores across categorical variables. When ANOVA results were significant, post hoc Tukey tests were applied [21]. For variables violating the assumption of homogeneity of variance, Welch’s ANOVA was used, followed by Games–Howell post hoc testing as appropriate.

Adequate sleep knowledge (≥60%) was analyzed as a binary outcome using logistic regression. Univariable logistic regression was first performed, and variables showing statistical significance or considered conceptually important were entered into a multivariable logistic regression model. Logistic regression model assumptions were evaluated prior to analysis. Multicollinearity among predictors was assessed conceptually and by inspecting standard errors, and highly correlated variables were not entered simultaneously into multivariable models. The number of predictors included in the final multivariable model was deliberately limited relative to the number of outcome events to reduce the risk of overfitting. Given the sample size and outcome frequency, formal goodness-of-fit testing and extensive residual diagnostics were not emphasized, and regression estimates were interpreted cautiously with attention to confidence intervals rather than sole reliance on statistical significance. Internal consistency reliability of the ASKME instrument in the present sample was assessed using Cronbach’s alpha computed across the 30 dichotomously scored items (correct = 1; incorrect/“Don’t know” = 0). All statistical analyses were conducted using Stata version 16 (StataCorp, College Station, TX, USA), with a two-sided *p*-value < 0.05 considered statistically significant.

### 4.9. Informed Consent and Ethical Considerations

Electronic informed consent was obtained from all participants. The first page of the online questionnaire served as an information sheet outlining the study objectives, voluntary nature of participation, and assurances of anonymity and confidentiality. To ensure anonymity, demographic information collection included a “Prefer not to say” option. Participants were required to select an “I consent” option to proceed with the questionnaire; selecting “I do not consent” resulted in automatic termination of the questionnaire. Total time to complete the questionnaire was recorded to ensure that no manipulation took place.

## 5. Conclusions

This study provides the first systematic assessment of sleep and sleep disorder knowledge among physicians working within Qatar’s PHCC using a validated instrument. Overall sleep knowledge was suboptimal, with fewer than half of participants achieving adequate sleep knowledge, and marked deficits were observed in clinically relevant domains, particularly common sleep disorders and sleep architecture. Among the variables examined, years of clinical experience was the only factor independently associated with higher sleep knowledge. These findings establish a baseline characterization of sleep medicine knowledge within Qatar’s public primary care system. As this study assessed knowledge rather than clinical behavior, the findings should be viewed as identifying educational gaps rather than demonstrating direct effects on patient care.

## Figures and Tables

**Table 1:** Baseline characteristics of PCPs included.

Variables Collected	Category	Frequency	Percentage
Age	20–29 years	1	0.91
	30–39 years	26	23.64
	40–49 years	36	32.73
	50–59 years	40	36.36
	60–69 years	7	6.36
Gender	Female	67	60.91
	Male	43	39.09
Nationality	Qatar	22	20.00
	Other Arab countries	27	24.55
	South Asian	1	0.91
	Western (Europe/North America)	24	21.82
	Prefer not to say	36	32.73
Country of university degree	Qatar	14	12.73
	Other Arab countries	30	27.27
	South Asian	14	12.73
	Western (Europe/North America)	11	10.00
	Prefer not to say	41	37.27
Specialty	Family Medicine	74	67.27
	General Practitioner	13	11.82
	Internal Medicine	4	3.64
	Community Medicine	8	7.27
	Dentistry	5	4.55
	Others	6	5.45
Years of experience	Less than 1 year	4	3.64
	1–5 years	16	14.55
	6–10 years	25	22.73
	11–15 years	23	20.91
	16–20 years	24	21.82
	More than 20 years	18	16.36
Degree	Basic medical degree only	26	23.64
	Board-certified physician	56	50.91
	Fellowship-trained	12	10.91
	Postgraduate academic degree (non-board)	16	14.55
Providing sleep-related counseling	No	40	36.36
	Yes	70	63.64
Received formal sleep training	66	60.00	66
	44	40.00	44
Source of sleep knowledge (multiple answers allowed)	Medical school curriculum	33	18.54
	Continuing Medical Education courses	14	7.87
	Conferences/workshops	12	6.74
	Online courses	23	12.92
	Self-study (e.g., books, journals)	44	24.72
	None	45	25.28
	Other	7	3.93
Most recent sleep knowledge update	Within the last year	3	2.73
	1–2 years ago	18	16.36
	3–5 years ago	22	20.00
	More than 5 years ago	21	19.09
	Never	46	41.82
Attending sleep-related continuing professional development activities	Yes, multiple times	9	8.18
	Yes, once	15	13.64
	No, but I am interested in future CPD activities	82	74.55
	No, and I am not interested in future CPD activities	4	3.64

**Table 2:** Percentage of correct answers per domain.

No.	Domian	Percentage of Correct Answers (Mean)	Std. Dev.	Min	Max
1	Basic sleep principles	63.6	22.05	12.50	100
2	Circadian sleep/wake control	72.9	22.27	0.00	100
3	Common sleep disorders	46.9	19.32	0.00	100
4	Effects of drugs and alcohol on sleep	49.1	29.85	0.00	100
5	Normal sleep architecture	49.7	23.89	0.00	100
	Overall percentage of correct answers	56.48	15.85	10.00	96.67
	Participants with ≥60% total score: 49/110	44.55			

**Table 3:** Comparison of mean ASKME scores across participant and clinical practice characteristics.

Variable	Category	Mean Score	SD	Frequency	Test	*p*-Value
Age group	20–29	53.33	0.00	1	One-way ANOVA	0.3399
	30–39	52.18	14.42	26		
	40–49	55.09	16.97	36		
	50–59	59.92	16.41	40		
	60–69	60.48	8.91	7		
Gender	Female	56.27	16.36	67	Independent samples *t*-test	0.8593
	Male	56.82	15.22	43		
Nationality (with “Prefer not to say” responses removed)	Qatari	60.00	16.46	22	One-way ANOVA	0.2333
	Other Arab	53.83	18.62	27		
	South Asian	56.67	0.00	1		
	Western	63.33	13.90	24		
Country of university degree (with “Prefer not to say” responses removed)	Qatari	52.62	15.64	14	One-way ANOVA	0.2044
	Other Arab	56.89	18.07	30		
	South Asian	61.19	15.06	14		
	Western	65.76	12.21	11		
Specialty	Family Medicine	57.21	15.65	74	One-way ANOVA	0.7550
	General Practitioner	56.92	12.87	13		
	Internal Medicine	51.67	5.77	4		
	Community Medicine	55.42	19.10	8		
	Dentistry	46.67	24.15	5		
	Subspeciality	59.44	19.25	6		
Years of experience	Less than 1 year	60.83	24.70	4	One-way ANOVA (violated equal variances; Bartlett’s test, *p* = 0.021)	0.0002
	1–5 years	48.13	16.33	16		
	6–10 years	53.47	18.27	25		
	11–15 years	51.01	10.51	23		
	16–20 years	59.72	10.49	24	Welch’s ANOVA	0.0015
	More than 20 years	69.81	13.36	18		
Degree	Basic degree only	53.85	14.04	26	One-way ANOVA	0.3297
	Board-certified	58.51	16.88	56		
	Fellowship-trained	59.17	15.12	12		
	Postgraduate degree	51.67	15.10	16		
Providing sleep-related counseling	No	55.83	12.12	40	Independent samples *t*-test	0.7462
	Yes	56.86	17.71	70		
Formal sleep training	No	55	15.13	66	Independent samples *t*-test	0.2306
	Yes	58.71	16.82	44		
Most recent sleep knowledge update	Within last year	54.44	5.09	3	One-way ANOVA	0.0325
	1–2 years	64.63	19.24	18		
	3–5 years	57.27	16.80	22		
	>5 years	59.84	13.76	21		
	Never	51.52	13.94	46		
Attended sleep-related CPD	Yes, multiple times	64.81	11.19	9	One-way ANOVA	0.2923
	Yes, once	54.67	13.26	15		
	No, but interested	56.30	16.04	82		
	No, not interested	48.33	26.74	4		

**Table 4:** Factors associated with adequate sleep knowledge (≥60%).

**Variable**	**Category**	**OR**	**SE**	***p*-Value**	**95% CI**
**Part A: Univariable Logistic Regression**					
Age group (ordinal categorical)		1.87	0.43	0.006	1.20–2.93
Gender (Male vs. Female)		0.84	0.33	0.650	0.39–1.81
Nationality (nominal categorical)	Qatar	1.00 (Reference)			
	Other Arab countries	0.35	0.21	0.081	0.11–1.14
	South Asian	1.00	(empty)		
	Western (Europe/North America)	2.50	1.59	0.150	0.72–8.71
	Prefer not to say	0.37	0.21	0.073	0.12–1.10
Country of university degree (nominal categorical)	Qatar	1.00 (Reference)			
	Other Arab countries	1.45	1.02	0.599	0.37–5.74
	South Asian	4.50	3.66	0.064	0.91–22.15
	Western (Europe/North America)	6.67	5.99	0.035	1.14–38.83
	Prefer not to say	1.77	1.19	0.395	0.48–6.60
Specialty (nominal categorical)	Family Medicine	1.00 (Reference)			
	General Practitioner	0.86	0.52	0.798	0.26–2.79
	Internal Medicine	0.34	0.39	0.351	0.03–3.35
	Community Medicine	0.34	0.28	0.196	0.06–1.76
	Dentistry	0.67	0.63	0.667	0.11–4.22
	Subspeciality	0.20	0.22	0.151	0.02–1.80
Years of experience (ordinal categorical)		1.86	0.30	<0.001	1.36–2.56
Degree (nominal categorical)	Basic medical degree	1.00 (Reference)			
	Board-certified physician	1.89	0.93	0.195	0.72–4.95
	Fellowship-trained	1.89	1.34	0.370	0.47–7.59
	Postgraduate academic degree	1.13	0.75	0.850	0.31–4.14
Providing sleep counseling (yes vs. no)		1.86	0.76	0.130	0.83–4.14
Formal sleep training (yes vs. no)		1.24	0.48	0.584	0.58–2.67
Most recent sleep knowledge update (ordinal categorical)		1.31	0.16	0.026	1.03–1.67
Attended sleep-related CPD (yes vs. no)		2.04	0.96	0.128	0.81–5.11
**Part B: Multivariable Logistic Regression (Including Strong Predictors and Conceptually Important Variables Only)**					
**Variable**	**Adjusted OR**	**SE**	***p*-Value**	**95% CI**	**Variable**
Gender	0.70	0.31	0.414	0.23–1.65	Gender
Years of experience	1.79	0.30	0.001	1.28–2.49	Years of experience
Most recent sleep knowledge update	1.21	0.16	0.144	0.94–1.57	Most recent sleep knowledge update
Attended sleep-related CPD	0.81	0.29	0.556	0.41–1.62	Attended sleep-related CPD

## Data Availability

The data presented in this study are only available on request from the corresponding author due to ethical restrictions related to participant confidentiality and the potential risk of indirect re-identification within a small, institution-specific study population.

## Abbreviations

The following abbreviations are used in this manuscript:

ANOVA	Analysis of Variance
ASKME	Assessment of Sleep Knowledge in Medical Education
CI	Confidence Interval
CPD	Continuing Professional Development
NREM	Non-Rapid Eye Movement
OR	Odds Ratio
PCPs	Primary Care Physicians
PHCC	Primary Health Care Corporation
REM	Rapid Eye Movement
SD	Standard Deviation
STROBE	Strengthening The Reporting of Observational Studies in Epidemiology
